# *Cdk8* and *Hira* mutations trigger X chromosome elimination in naive female hybrid mouse embryonic stem cells

**DOI:** 10.1007/s10577-024-09756-w

**Published:** 2024-10-10

**Authors:** Kevin Halter, Jingyi Chen, Tadeas Priklopil, Asun Monfort, Anton Wutz

**Affiliations:** 1 Institute of Molecular Health Sciences, Department of Biology, Swiss Federal Institute of Technology, ETH Hönggerberg, Zurich, Switzerland; 2 Department of Biology and Department of Environmental Systems Science, ETH Zurich, Zurich, Switzerland

**Keywords:** Hira, Cdk8, X chromosome elimination, Mouse embryonic stem cells, Naive pluripotency, Mus musculus castaneus

## Abstract

Mouse embryonic stem cells (ESCs) possess a pluripotent developmental potential and a stable karyotype. An exception is the frequent loss of one X chromosome in female ESCs derived from inbred mice. In contrast, female ESCs from crosses between different *Mus musculus* subspecies often maintain two X chromosomes and can model X chromosome inactivation. Here we report that combined mutations of *Hira* and *Cdk8* induce rapid loss of one X chromosome in a *Mus musculus castaneus* hybrid female ESC line that originally maintains two X chromosomes. We show that MEK1 inhibition, which is used for culturing naive pluripotent ESCs is sufficient to induce X chromosome loss. In conventional ESC media, *Hira* and *Cdk8* mutant ESCs maintain both X chromosomes. Induction of X chromosome loss by switching to naive culture media allows us to perform kinetic measurements for calculating the chromosome loss rate. Our analysis shows that X chromosome loss is not explained by selection of XO cells, but likely driven by a process of chromosome elimination. We show that elimination of the X chromosome occurs with a rate of 0.3% per cell per division, which exceeds reported autosomal loss rates by 3 orders of magnitude. We show that chromosomes 8 and 11 are stably maintained. Notably, *Xist* expression from one of the two X chromosomes rescues X chromosomal instability in Δ*Hira*Δ*Cdk8* ESCs. Our study defines mutations of *Hira* and *Cdk8* as molecular drivers for X chromosome elimination in naive female ESCs and describes a cell system for elucidating the underlying mechanism.

**Supplementary Information:**

The online version contains supplementary material available at 10.1007/s10577-024-09756-w.

## Introduction

The accumulation of cells with an XO karyotype in female mouse embryonic stem cells (ESCs) derived from inbred mouse strains has repeatedly been reported (Kawase et al. 1994; Rastan and Robertson 1985; Robertson et al. 1983; Keniry et al. 2022). Early experiments using mouse embryonal carcinoma cell lines already observed frequent acquisition of an XO karyotype, which was initially interpreted as Y chromosome loss in male cells (Robertson et al. 1983). Later studies establishing ESC lines directly from mouse embryos, without the need for teratocarcinoma formation (Martin 1981; Evans and Kaufman 1981), showed that the large majority of XO karyotypes originated from female ESCs and suggested an instability of the X chromosome in culture (Robertson et al. 1983). Loss of one X chromosome in female ESC cultures from inbred mouse strains has been reported as early as 1983 (Robertson et al. 1983) and remains an enigmatic observation, which has received scant attention to date owing to a lack of a tractable experimental system. Accordingly, the causes for the instability of XX karyotypes have remained elusive. Potential explanations have been put forward including the idea of selection for XO cells in culture driven by an increased fitness compared to ESCs with elevated X-linked gene dosage (Rücklé et al. 2023). Observations of a hypomethylated genome in XX ESCs compared to XY and XO ESCs has also been considered a potentially contributing factor (Zvetkova et al. 2005; Ooi et al. 2010). It has further been shown that genome-wide DNA hypomethylation can lead to chromosomal instability (Sheaffer et al. 2016; Pappalardo and Barra 2021), which might be involved in X chromosome loss. However, experimental support for any mechanism remains limited to date.

Advances in the understanding of mouse embryogenesis have facilitated improvements of culture conditions for mouse ESCs. Conversely, media formulations have been developed that can approximate specific states of pluripotency (Ying et al. 2008). In particular, supplementation of culture media with small molecule inhibitors of the MEK1 and GSK3 protein kinases (Tamm et al. 2013; Ying et al. 2008) has been used to induce a ground state of pluripotency in naive ESC cultures that resembles characteristics of early epiblast cells. The MEK1 inhibitor PD0325901 (Barrett et al. 2008) and the GSK3 inhibitor CHIR99021 are included in 2i medium formulations for naive ESC cultures (Marks et al. 2012). ESCs in 2i medium show reduced DNA methylation compared to conventional cultures in serum and leukemia inhibitory factor (LIF) based media (Yagi et al. 2017; Choi et al. 2017).

Female mouse ESCs are widely used as a model for studying X chromosome inactivation (XCI), which is the process that establishes X chromosomal dosage compensation between the XX female and XY male karyotypes in mammals (Wutz and Gribnau 2007). It has been shown that female ESCs initiate silencing of one X chromosome when induced to differentiate in culture (Martin et al. 1978). Initiation of XCI is regulated by the presence of two X chromosomes and is not observed in differentiating XO or male XY ESCs (Rastan and Robertson 1985). Female mouse ESC lines obtained from hybrid crosses between lab mice and different subspecies of *Mus musculus*, in particular *Mus musculus castaneus* (Chou et al. 1998) and *Mus musculus molossinus* (Okumura et al. 2021) are preferentially used in the X inactivation field and have been observed to maintain two X chromosomes (Tarruell Pellegrin 2019).

We have previously identified cyclin dependent kinase 8 (*Cdk8*) and histone cell cycle regulator A (*Hira*) as candidate silencing factors for XCI by forward genetic screening in haploid ESCs (Monfort et al. 2015). Subsequent analysis has shown that loss of *Cdk8* causes a partial defect in repression of X-linked genes at the initiation of XCI (Postlmayr et al. 2020), raising the question if *Hira* and *Cdk8* might act in parallel pathways in XCI. *Cdk8* is a member of the family of cyclin dependent kinases that require cyclins for their catalytic function (Nemet et al. 2014). CDK8 associates with cyclin C, and the mediator complex components MED12 and MED13 forming a kinase submodule (Fant and Taatjes 2019), which regulates gene expression. It has been shown that *Cdk8* inhibitors or overexpression of catalytically inactive *Cdk8* can induce naive pluripotent characteristics in ESCs (Lynch et al. 2020). HIRA associates with UBN1 and CABIN1 to form a chromatin assembly complex (Ray-Gallet et al. 2018), which deposits the histone H3 variant H3.3 independent of DNA replication. *Hira* is required for histone H3.3 integration in transcriptionally active chromatin and gene regulatory elements (Szenker et al. 2011). In heterochromatic domains at centromeres and telomeres histone H3.3 deposition is independent of *Hira*, but requires a different histone chaperone complex containing ATRX and DAXX (Dyer et al. 2017). Thus, *Hira* and *Cdk8* associate with and contribute to regulating expression of genes through biochemically distinct complexes.

Here we investigate overlapping functions of *Cdk8* and *Hira* in ESCs by engineering combined *Cdk8* and *Hira* mutations in a female Cast/Ei hybrid ESC line that stably maintains two X chromosomes. Unexpectedly, we encountered rapid accumulation of XO cells in *Hira*, Cdk8 double mutant (Δ*Hira*Δ*Cdk8*) ESCs, which precluded an analysis of XCI. We show that chromosome loss is specific for the X chromosome and associated with MEK1 inhibition, suggesting a feature of ground state pluripotency. Kinetic analysis shows that X chromosome loss cannot be explained by selection of XO cells leading us to propose a process of chromosome elimination.

## Results

### X chromosome instability in naive Δ*Hira*Δ*Cdk8* female hybrid ESCs

**Fig. 1:**
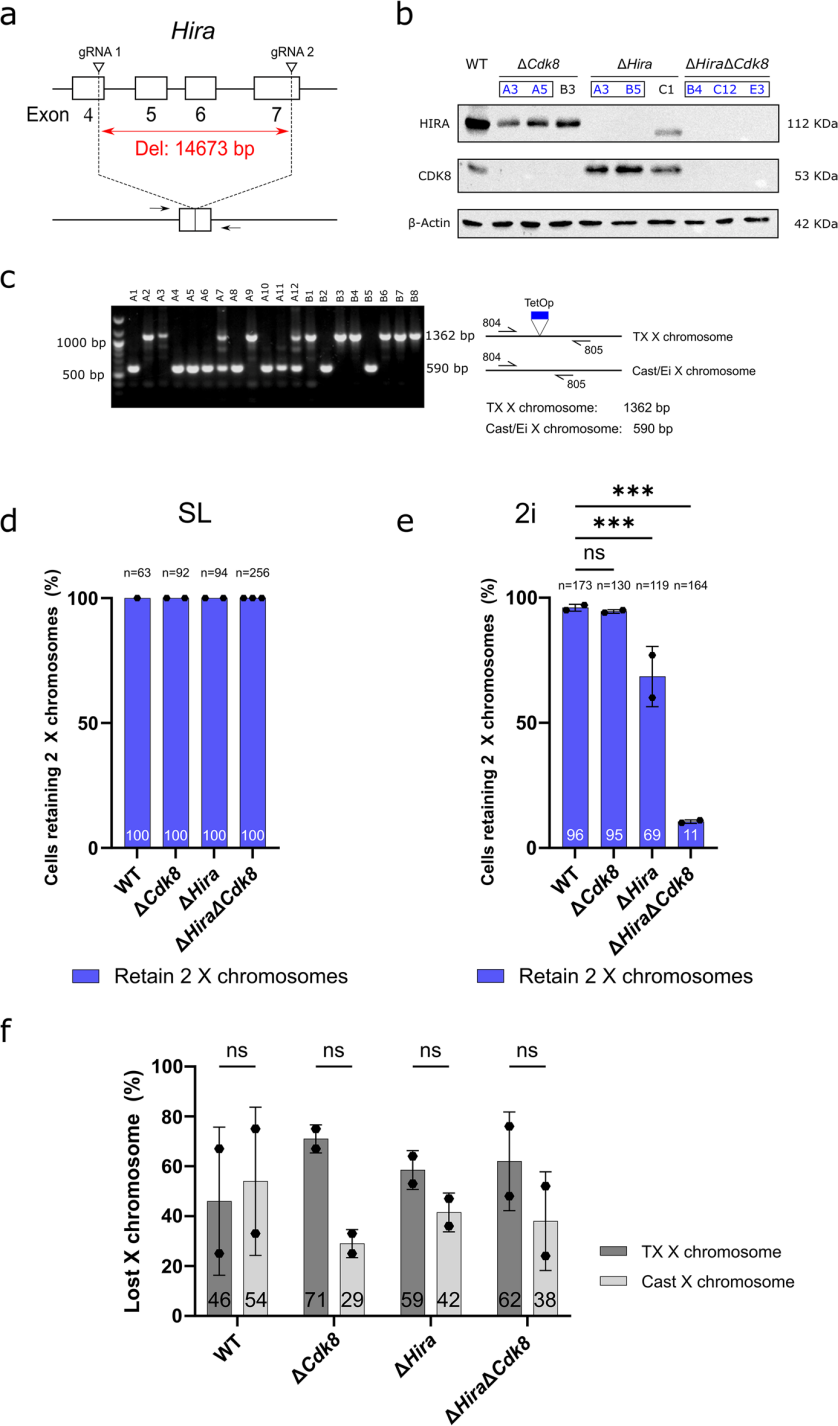
X elimination is media and genotype dependent. **a** *Hira* knockout strategy resulting in the complete excision of exons 5, 6 and the partial deletion of exons 4 and 7. **b** Western blot confirming the successful gene deletions in *Cdk8* and *Hira* single as well as double mutants. Cell lines indicated with blue text and box were used in this study. **c** Left: Representative gel electrophoresis results of the allelic PCR with primers 804 and 805 for Δ*Hira*Δ*Cdk8* B4 in the 2i condition. Right: Schematic overview of the allelic PCR used to visualize the presence of TX and Cast/Ei X chromosomes. **d, e** Allelic PCR screen results indicating the percentage of screened colonies retaining two X chromosomes in the different genotypes and media conditions after 2 weeks. Data is shown as mean ± SD. Individual dots correspond to biological replicates, n indicates the number of screened colonies. Biological replicates showed no statistically significant effect, and hence we combined the data and used Pearson’s Chi-squared test with Yates’ continuity correction, which showed a significant association between genotype and X chromosome loss between WT and *Hira* mutant X^2^(1, *N* = 292) = 31.481, *p* < 2.2e-16; a significant association between genotype and X chromosome loss between WT and *Hira*,*Cdk8* double mutant X^2^(1, *N* = 337) = 245.09, *p* = 2.014e-08; but no significant association between genotype and X chromosome loss between WT and *Cdk8* mutant X^2^(1, *N* = 303) = 0.074423, *p* = 0.785. **f** Bar graph showing the allele specific distribution of lost X chromosomes of Δ*Hira*Δ*Cdk8* ESCs in 2i medium following the allelic PCR screen. Data is shown as mean ± SD**.** To account for variability between replicates, a generalized linear mixed model with a binomial distribution was used to investigate the effect of genotypes on maternal and paternal X chromosome loss frequencies. The results indicate that the baseline probability of the outcome for genotype as not significantly different from 0.5. Indicating that maternal and paternal X chromosomes are lost in a random manner. WT (intercept) β = 0.2490, 95% CI [-1.3656, 1.8636] *p* = 0.762; *Hira* mutant β = -0.5135, 95% CI [-2.1928, 1.1659] *p* = 0.549; *Cdk8* mutant β = -1.2447, 95% CI [-3.5005, 1.0110], *p* = 0.279; *Hira*,*Cdk8* double mutant β = -0.7497, 95% CI [-2.3106, 0.8111], *p* = 0.346

For investigating overlapping functions of *Cdk8* and *Hira* we established a female ESC line from a cross of female TX mice, which carry a doxycycline inducible *Xist* allele on a mixed C57BL/6 and 129 background (Savarese et al. 2006), and inbred *Mus musculus castaneus* Cast/Ei males (Roderick 1982). Our ESC line maintained a stable karyotype of 40 chromosomes including two X chromosomes in 2i medium. We introduced *Cdk8* and *Hira* mutations by transient transfection of CRISPR/Cas9 nuclease expression vectors (Fig. 1a, b), the *Cdk8* deletion strategy has previously been described (Postlmayr et al. 2020). Although, we isolated several colonies with homozygous mutations in both *Hira* and *Cdk8*, few of the Δ*Hira*Δ*Cdk8* ESC subclones maintained two X chromosomes. In most cases we observed an XO karyotype with complete loss of either the TX or Cast/Ei X chromosome. In contrast, several ESC subclones with either a Δ*Hira* or a Δ*Cdk8* mutation maintained a normal karyotype of 40 chromosomes including two X chromosomes. In order to explore if Δ*Hira*Δ*Cdk8* ESCs with two X chromosomes could be reliably established, we investigated different culture media. We finally were able to obtain Δ*Hira*Δ*Cdk8* ESC subclones in conventional ESC medium based on serum and LIF (SL) that maintained both TX and Cast/Ei X chromosomes. Our results suggested that 2i medium might have triggered the loss of one X chromosomes in Δ*Hira*Δ*Cdk8* ESCs.

For quantifying X chromosome loss we isolated single-cell subclones of wildtype control (WT), Δ*Hira*, Δ*Cdk8*, and Δ*Hira*Δ*Cdk8* ESCs in SL conditions by colony picking. For each genotype at least two biological replicates were used. Subsequently, the presence of both X chromosomes was confirmed by PCR (Fig. 1c), using primers flanking the Tet operator insertion on the TX X chromosome. The PCR product from the TX chromosome differs in size from the Cast/Ei wild type *Xist* allele, which does not harbour the Tet operator. Subclones were split and then cultured in SL and 2i medium for 2 weeks. Subsequently, we isolated subclones through colony picking and analysed the presence of the two X chromosomes by Tet operator PCR. We observed that TX/Cast wildtype (63/63), Δ*Cdk8* (92/92), Δ*Hira* (94/94), and Δ*Hira*Δ*Cdk8* (256/256) ESC subclones maintained both X chromosomes after 2 weeks of culture in SL medium (Fig. 1d) confirming a remarkable stability of the X chromosomes. In parallel cultures in 2i medium, TX allele specific PCR showed the presence of two X chromosomes in 96% and 95% of WT and Δ*Cdk8* subclones, respectively (Fig. 1e). In contrast, only 69% of Δ*Hira* subclones maintained both X chromosomes indicating a measurable X chromosome loss. The most striking difference between SL and 2i media was observed for Δ*Hira*Δ*Cdk8* ESCs. Whereas both X chromosomes were stably maintained in SL conditions, only 11% of 164 subclones of Δ*Hira*Δ*Cdk8* ESCs maintained both X chromosomes in 2i medium. Therefore, combined mutations of *Hira* and *Cdk8* caused significant X chromosome loss in 2i but not SL medium. Either the maternal TX or paternal CAST/EI X chromosome were lost and we did not observe a statistically significant difference (Fig. 1f).

### Chromosome loss is X chromosome specific and occurs with fast kinetics in naive Δ*Hira*Δ*Cdk8* ESCs

**Fig. 2:**
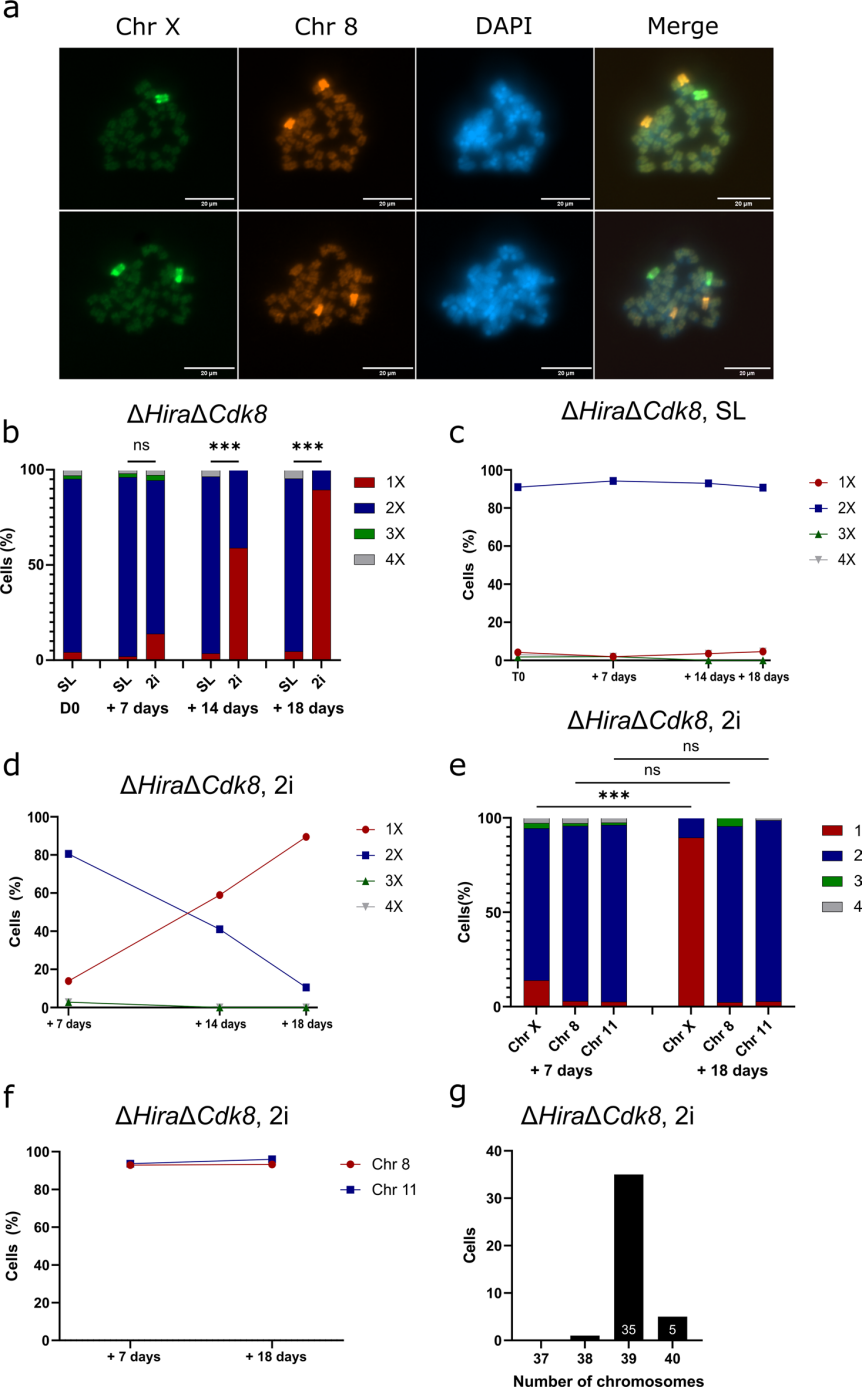
Time course experiments reveal the fast kinetics of X elimination and show that autosomes are stably maintained. **a** Representative DNA FISH image with probes for chromosomes 8 (orange) and X (green). Scale bar corresponds to 20 µm. **b, c, d** Characterization of X chromosomal elimination in Δ*Hira*Δ*Cdk8* ESCs by DNA FISH. Graphs show percentage of cells retaining the indicated number of X chromosomes in the different media conditions at consecutive time points. We used multinomial logistic regression to measure the effect of the culture medium (SL vs 2i) on the X chromosomal status. Day 7: The model reports a coefficient of 2.134 for the 1X phenotype in 2i medium, this effect is not statistically significant with a Wald test *p* = 0.0568 and 95% CI [-0.062, 4.330] but indicates a trend towards significance. Day 14: The 2i medium significantly influenced the 1X phenotype (Coefficient = 3.638162, 95% CI [2.130, 5.146], *p* = 2.29e-06. Day 18: The 2i medium significantly influenced the 1X phenotype (Coefficient = 5.115593, 95% CI [3.883, 6.348], *p* = 4.440892e-16). **e** DNA FISH results showing the change in the indicated number of chromosomes X, 8 and 11 in Δ*Hira*Δ*Cdk8* ESCs during culture in 2i medium. Fisher’s Exact tests were used to analyse the change in chromosome counts from day 7 to day 18. Chromosome X yields a highly significant result with *p* = 1.178e-13, indicating significant change from day 7 to day 18. Chromosomes 8 (*p* = 0.2993) and 11 (*p* = 1) show no significant change from day 7 to day 18. **f** Line graph showing the change in the fraction of Δ*Hira*Δ*Cdk8* cells maintaining 2 versions of chromosomes 8 and 11 during prolonged culture in 2i medium. **g** Karyotyping of Δ*Hira*Δ*Cdk8* ESCs cultured in 2i medium for 21 days. Bar graph shows the number of analysed cells containing the indicated number of chromosomes

To investigate X chromosomal instability further, we performed DNA FISH analysis using X chromosome specific paint probes (Fig. 2a, S1a). We cultured clonal populations of Δ*Hira*Δ*Cdk8* ESCs in SL medium and observed two X chromosomes in 91% of Δ*Hira*Δ*Cdk8* ESCs. The starting cultures were subsequently split into SL and 2i medium and fixed on slides after 7, 14 and 18 days to perform counting of X chromosomes (Fig. 2b, c, d). After one week in 2i medium the fraction of cells with two X chromosomes declined to 81%, while in SL medium 94% of the cells showed two X chromosomes. Strikingly, after two weeks of 2i culture, the majority of Δ*Hira*Δ*Cdk8* ESCs had acquired an XO karyotype, with only 41% of cells retaining both X chromosomes. In SL medium 93% of cells maintained two X chromosomes at this time point. At the last time point, after 18 days of culture in 2i medium only 11% of Δ*Hira*Δ*Cdk8* ESCs retained an XX karyotype. In contrast, X chromosomes were stable in SL medium with 91% of the cells showing two X chromosomes. Our time course demonstrated a rapid accumulation of the XO karyotype in 2i medium raising the possibility that in ground state pluripotency an unknown process triggers chromosome instability in the absence of *Hira* and *Cdk8*.

We next investigated if instability was specific for X chromosomes or also affected autosomes. Previously, trisomies of chromosome 8 and 11 have been observed to accumulate in ESC cultures (Gaztelumendi and Nogués 2014). We reasoned, that in case autosomal monosomies might be selected against, we would nonetheless be able to observe gains of chromosome 8 or 11, if numeric karyotype aberrations were the result of non-disjunction events in naive Δ*Hira*Δ*Cdk8* ESCs. We performed DNA FISH with specific paint probes for either chromosome 8 or 11. These Δ*Hira*Δ*Cdk8* ESCs lost X chromosomes in 2i medium, and the fraction of cells with an XO karyotype reached 89% after 18 days. In contrast, we observed two copies of chromosome 8 and 11 in 93% and 96% of Δ*Hira*Δ*Cdk8* ESCs, respectively (Fig. 2e, f). Therefore, autosomes 8 and 11 were stably maintained, showing no significant change in cells displaying significant X chromosomal loss during the same time period.

**Fig. 3:**
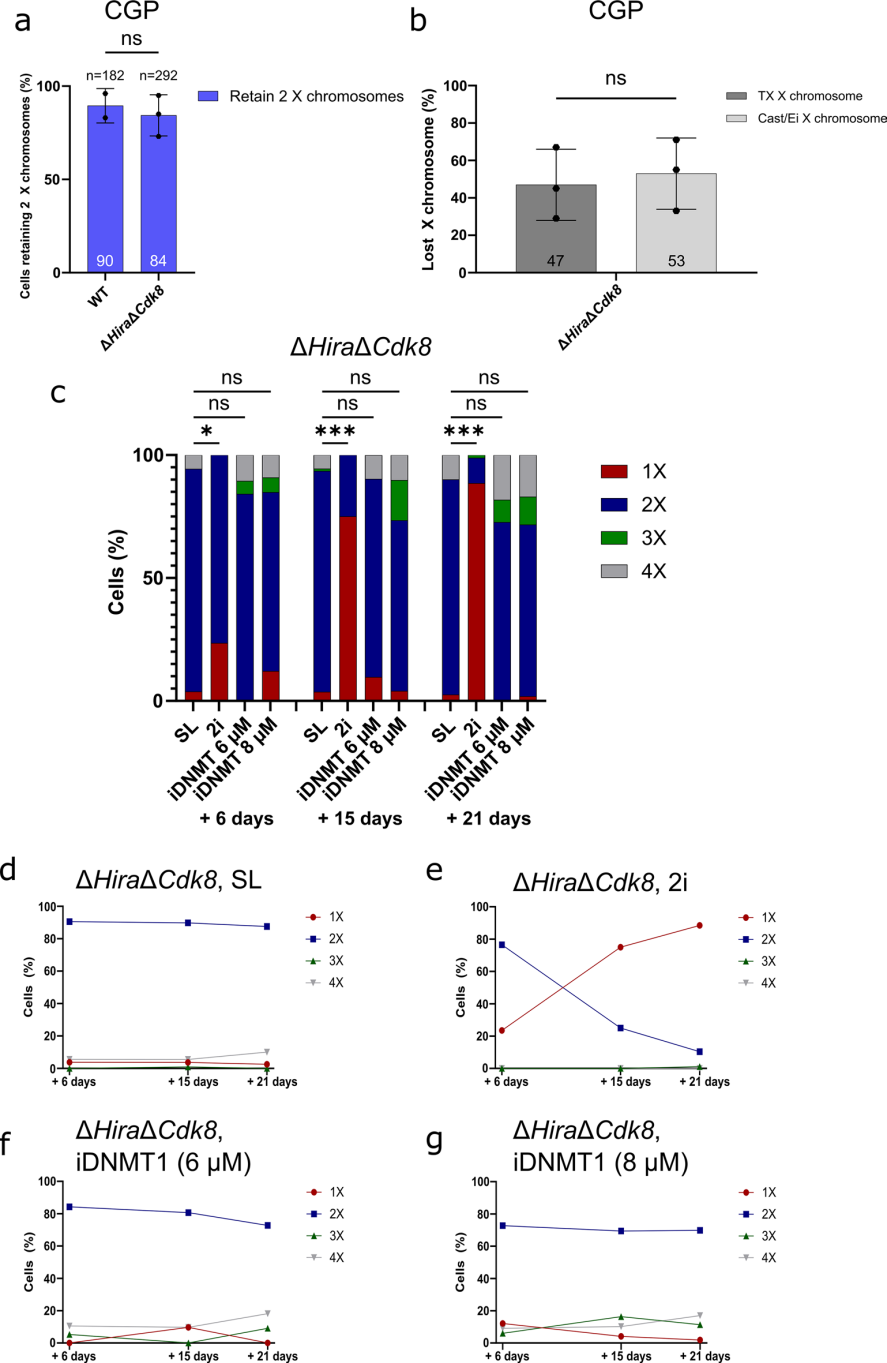
Induced hypomethylation alone does not lead to X elimination in Δ*Hira*Δ*Cdk8* ESCs. **a** Characterization of X chromosomal stability in CGP medium. Bar graph shows the percentage of analysed cells of wildtype control (WT) and Δ*Hira*Δ*Cdk8* ESCs retaining two X chromosomes in CGP medium, n indicates the number of screened colonies/cells. Individual data points correspond to biological replicates. Data is shown as mean ± SD. Mixed effects logistic regression model indicates no statistically significant effect of genotype on X chromosomal status (Treating replicates as random effects, the estimated effect of the double mutant genotype is –0.28, 95% CI [-0.82. 0.26], z = -1.03, *p* = 0.303). An additional Chi-squared test was used to compare the distribution of phenotypes between genotypes and found no significant association (X^2^ = 0.81, df = 1, *p* = 0.369). **b** Bar graph showing the allele specific distribution of lost X chromosomes for Δ*Hira*Δ*Cdk8* ESCs in CGP medium. Individual points correspond to biological replicates. Data is shown as mean ± SD. Exact binomial tests shows no significant deviation from 0.5 probability of success for all replicates. (Replicate 1: observed probability of success = 0.55, 95% CI [0.315, 0.769], *p* = 0.8238; Replicate 2: observed probability of success = 0.708, 95% CI [0.489, 0.874], *p* = 0.06391; Replicate 3: observed probability of success = 0.333, 95% CI [0.008, 0.906], *p* = 1). **c** Characterization of X chromosomal elimination in Δ*Hira*Δ*Cdk8* ESCs cultured in SL, 2i and iDNMT1 6 µM and µ8 M (SL + 6 and 8 µM GSK3484862) media conditions by DNA FISH. Bar graph shows the percentage of cells containing the indicated number of X chromosomes per media condition after 6, 15 and 21 days of culture. Multinomial logistic regression analysis found that across all time points 2i medium significantly increased the 1X phenotype compared to the baseline SL medium. iDNMT 6 µM and iDNMT 8 µM media how no significant effects on the 1X phenotype compared with SL. (Day 6: 2i: Coefficient of 2.00, 95% CI [0.19, 3.81], *p* = 0.0299; iDNMT 6 µm: Coefficient of -8.07, 95% CI [-144.93, 128.78], *p* = 0.9073; iDNMT 8 µM: Coefficient of 1.39, 95% CI [-0.38, 3.16], *p* = 0.1239. Day 15: 2i: Coefficient of 4.29, 95% CI [3.19, 5.39], *p* = 2.398e-14; iDNMT 6 µm: Coefficient of 1.07, 95% CI [-0.49, 2.63], *p* = 0.1795; iDNMT 8 µm Coefficient of 0.36, 95% CI [-1.37, 2.08], *p* = 0.6893. Day 21: 2i: Coefficient of 5.70, 95% CI [3.59, 7.82], *p* = 1.089e-07; iDNMT 6 µm: Coefficient of -10.85, 95% CI [-547.68, 526.98], *p* = 0.9684; iDNMT 8 µm: Coefficient of -0.06, 95% CI [-2.87, 2.76], *p* = 0.9689. **d, e, f**, **g** Line graphs visualizing the change in X chromosomal composition over time in Δ*Hira*Δ*Cdk8* ESCs

We furthermore performed karyotyping of Δ*Hira*Δ*Cdk8* ESCs after 21 days in 2i culture (Fig. 2g, S1b). Of 41 metaphase spreads, 35 (85%) showed 39 chromosomes, 5 (12%) 40 chromosomes, and 1 (2%) 38 chromosomes. The 85% metaphase spreads with 39 chromosomes closely match our observation of 88% XO cells by DNA FISH analysis of the same sample (Fig. 3c). Taken together our analysis demonstrated that autosomes were stably maintained in the majority of Δ*Hira*Δ*Cdk8* ESCs. In contrast X chromosome loss occurred rapidly in 2i culture.

### MEK1 inhibition is sufficient to induce X chromosomal loss in Δ*Hira*Δ*Cdk8* ESCs

Our results showed that culture in 2i medium alone was insufficient to induce X chromosomal instability in hybrid ESCs, and over 90% of wild type hybrid TX/Cast ESCs maintained two X chromosomes over two weeks. Similarly, Δ*Hira*Δ*Cdk8* ESCs maintained two X chromosomes in conventional SL culture. However, culture in 2i medium induced rapid X chromosome loss specifically in Δ*Hira*Δ*Cdk8* ESCs with 89% of cells becoming XO after 2 weeks.

To further investigate if a specific component of 2i medium or a general effect of ground state pluripotency triggers X chromosome instability we took advantage of different culture media for naive ESCs. A combination of SRC and GSK3 kinase inhibition has been shown previously to support ESC cultures in a similar way as 2i (Di Stefano et al. 2018). Notably, this study replaced MEK1 for SRC inhibition requiring only a change of a single component in the media formulation. We analysed X chromosomal stability of wildtype control and Δ*Hira*Δ*Cdk8* ESCs in medium containing the SRC inhibitor CGP 77675, GSK3 inhibitor CHIR99021 and LIF, henceforth this medium is called CGP. Wildtype control ESCs cultured in CGP medium for 2 weeks show a stable X chromosomal constitution with 90% of screened colonies retaining two X chromosomes averaged across the 2 replicates. Δ*Hira*Δ*Cdk8* cells cultured in CGP medium for 2 weeks also remain X chromosomally stable with 84% of screened colonies retaining 2 X chromosomes averaged across the three biological replicates (Fig. 3a). In comparison, parallel culture of Δ*Hira*Δ*Cdk8* cells in 2i medium led to drastic X chromosomal loss with only 11% of screened colonies retaining both X chromosomes (Fig. 1e). In both CGP and 2i medium either the maternal TX X chromosome or the paternal Cast/Ei X chromosome were lost (Fig. 3b, 1f).

**Fig. 4:**
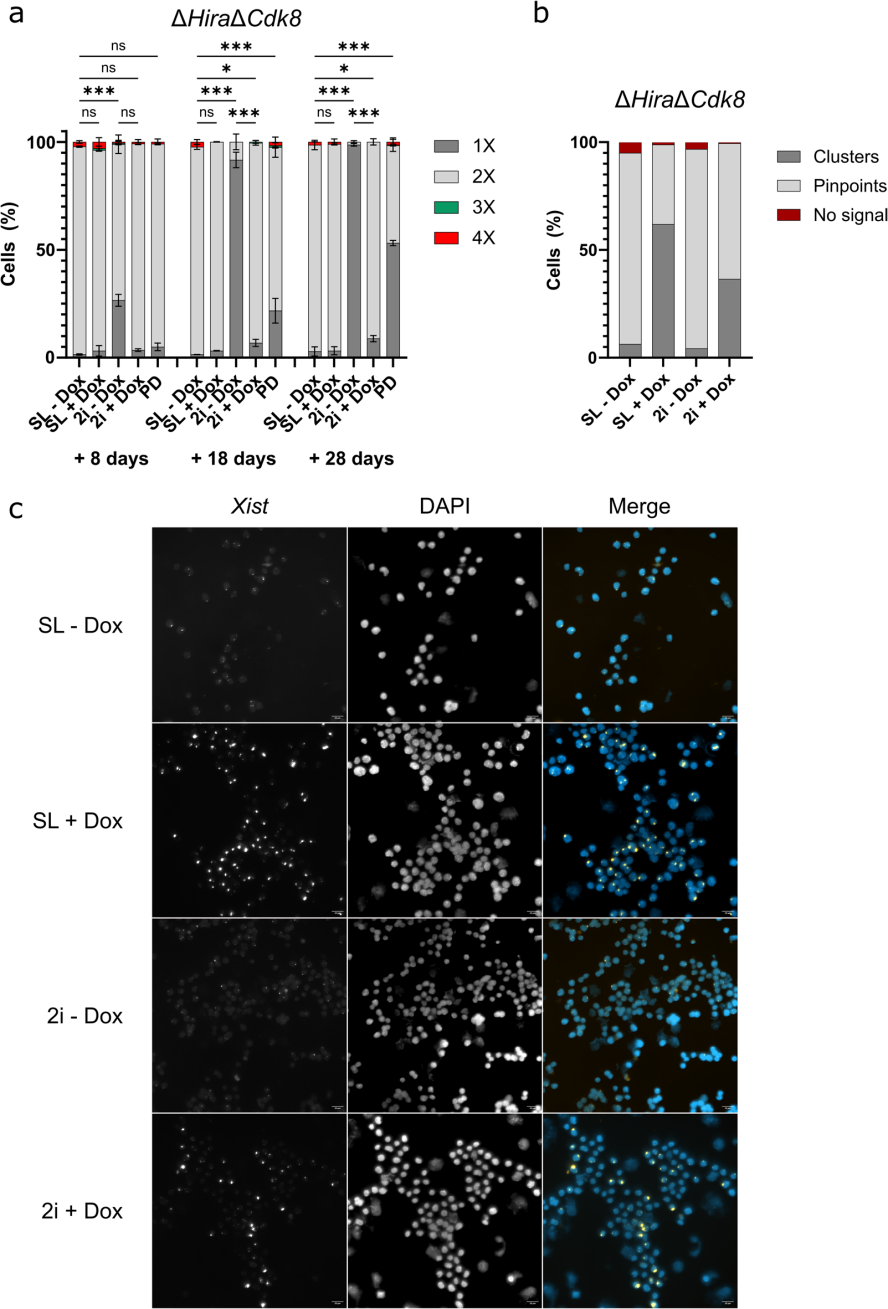
Induced *Xist* expression rescues X chromosomal instability in Δ*Hira*Δ*Cdk8* ESCs. **a** Time-course analysis of X chromosomal loss in Δ*Hira*Δ*Cdk8* ESCs cultured in SL - Dox, SL + Dox, 2i - Dox, 2i + Dox and PD medium. Bar graph shows the percentage of cells containing the indicated number of X chromosomes per media condition after 8, 18 and 28 days of culture. Data is shown as mean ± SD of two independent Δ*Hira*Δ*Cdk8* ESC clones. We used a multinomial logistic regression model to assess the effects of the different media conditions on the 1X phenotype across the different time points, using the SL – Dox condition as the reference group. The 2i—Dox consistently showed a highly significant increase of the 1X fraction at each time point (Day 8: Estimate = 3.39, 95% CI [2.06, 5.37], *p* < 0.001; Day 18: Estimate = 6.87, 95% CI [5.50, 8.86], *p* < 0.001; Day 28: Estimate = 8.06, 95% CI [6.64, 9.68, *p* < 0.001). The 2i + Dox showed a barely significant increase of the 1X fraction at later time points (Day 8: Estimate = 0.96, 95% CI [-0.75, 3.16], *p* > 0.05; Day 18: Estimate = 1.79, 95% CI [0.41, 3.77], *p* < 0.05; Day 28: Estimate = 1.19, 95% CI [0.13, 2.31], *p* < 0.05). PD medium showed a highly significant increase at day 18 and 28, but not on day 8 (Day 8: Estimate = 1.39, 95% CI [-0.19, 3.37], *p* > 0.05; Day 18: Estimate = 3.16, 95% CI [1.81, 5.16], *p* < 0.001; Day 28: Estimate = 3.69, 95% CI [2.74, 4.74], *p* < 0.001). SL + Dox did not show a significant increase of the 1X fraction at any time point (Day8: Estimate = 0.87, 95% CI [-0.82, 3.03], *p* > 0.05; Day 18: Estimate = 0.90, 95% CI [-0.86, 3.05], *p* > 0.05; Day 28: Estimate = 0.10, 95% CI [-1.35, 1.48], *p* > 0.05). Comparing 2i – Dox to 2i + Dox at each time point, we report a highly significant difference on Day 18 (Estimate difference = 5.08, 95% CI [2.75, 7.41], *p* < 0.001) and Day 28 (Estimate difference = 6.87, 95% CI [4.99, 8.75], *p* < 0.001), but not on Day 8 (Estimate difference = 2.43, 95% CI [-0.06, 4.92], *p* = 0.056). **b** Quantification of *Xist* clusters and pinpoint signals in the indicated media conditions. **c** Representative images of RNA FISH with *Xist* specific probes of Δ*Hira*Δ*Cdk8* ESCs cultured in SL – Dox, SL + Dox, 2i – Dox and 2i + Dox medium. Scale bar corresponds to 20 µm

To investigate if MEK1 inhibition by PD03 is sufficient to induce X chromosomal loss, we performed a time course experiment with two independent Δ*Hira*Δ*Cdk8* ESC clones (Fig. 4a). Cells were first grown in SL medium and then split into SL, 2i and PD (PD03 + LIF) medium, X chromosomal status was assessed after 8, 18 and 28 days of culture by DNA FISH and chromosome counting. Δ*Hira*Δ*Cdk8* ESCs cultured in SL medium showed high X chromosomal stability across all time points, with 97% (56/58) and 96% (74/77) of analysed cells retaining 2 X chromosomes after 8 days for the respective replicates, 97% (65/67) and 95% (62/65) after 18 days, 97% (70/72) and 94% (64/68) after 28 days. Cells cultured in PD medium showed a gradual decrease of the 2X fraction and an increase of the 1X fraction. After 8 days 94% (50/53) and 94% (60/64) of analysed cells showed two X chromosomes, this decreased to 72% (42/58) and 79% (49/62) after 18 days and to 43% (32/74) and 47% (92/195). Cells cultured in 2i medium showed rapid X chromosome loss with the 2X fraction decreasing from 69% (63/91) and 75% (58/77) at day 8, to 6% (5/86) and 11% (12/110) at day 18, to 0.9% (1/107) and 1.75% (2/114) at day 28. These results show that X chromosome loss in PD medium is substantially elevated compared to SL conditions, but does not reach the levels seen in the full 2i medium.

Our results demonstrated that MEK1 inhibition leads to rapid X chromosome loss in naive female Δ*Hira*Δ*Cdk8* ESCs in 2i medium, but naive cultures using SRC and GSK3 inhibitors show higher levels of X chromosomal stability. Additionally, we show that MEK1 inhibition by PD03 is sufficient to induce X chromosomal loss.

Although both MEK1 and SRC inhibition induce ground state pluripotency in combination with GSK3 inhibition, differences in the level of DNA methylation have been noted (Yagi et al. 2017). In 2i cultures DNA methylation decreases to a level that is comparable to the preimplantation epiblast (Yagi et al. 2017). In contrast, DNA methylation in presence of SRC and GSK3 inhibition appears at an intermediate level between SL and 2i culture. To determine if loss of DNA methylation might be the trigger of X chromosome loss in 2i medium, we made use of a chemical inhibitor of DNMT1. GSK3484862 has been shown to mediate dramatic DNA demethylation in mouse ESCs without compromising cell viability (Azevedo Portilho et al. 2021). We performed DNA FISH for counting the number of X chromosomes in Δ*Hira*Δ*Cdk8* ESCs cultured in SL medium supplemented with DNMT1 inhibitor GSK3484862 (from here on referred to as iDNMT1). ESCs cultured in 2i and SL served as positive and negative controls for X chromosome instability. We observed no significant X chromosomal loss in cells cultured with either 6 µM or 8 µM of DNMT1 inhibitor after 6, 15 or 21 days (Fig. 3c, f, g). In contrast, 2i medium induced rapid accumulation of an XO karyotype in 24%, 75%, and 89% of ESCs after 6, 15, and 21 days of culture, respectively (Fig. 3c, e), which is consistent with our earlier observations. As expected Δ*Hira*Δ*Cdk8* ESCs cultured in SL medium without any inhibitors showed no significant accumulation of cells with an XO karyotype (Fig. 3c, d). Our data, thus, suggest that loss of DNA methylation alone is insufficient to induce X chromosome loss in female Δ*Hira*Δ*Cdk8* ESCs but additional effects of MEK1 or GSK3 inhibition are also required.

### Kinetics of X chromosome loss in naive Δ*Hira*Δ*Cdk8* ESCs is not explained by proliferative advantage of the XO karyotype

**Fig. 5:**
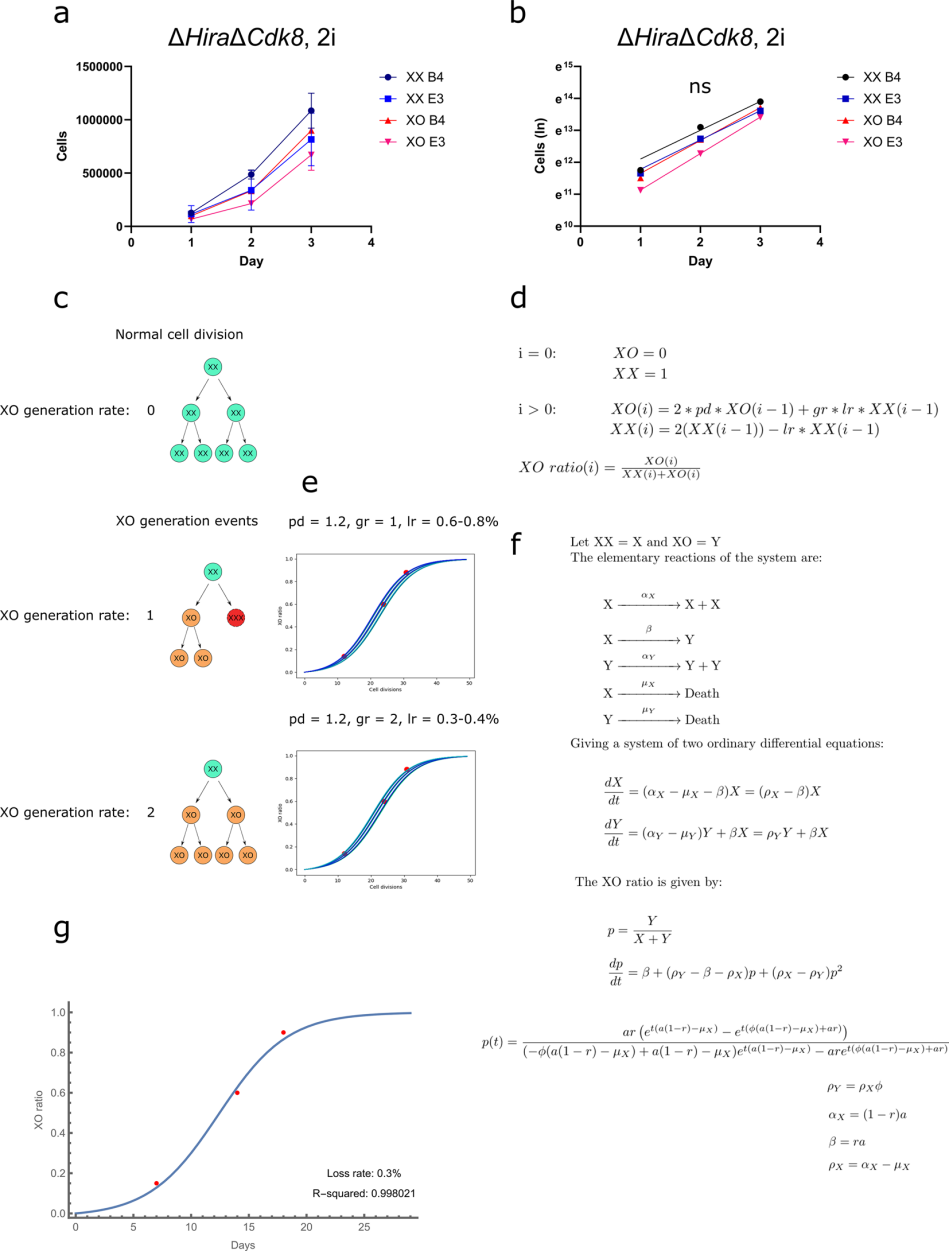
Mathematical modelling reveals a high X chromosomal loss rate. **a** Proliferation curves of XX and XO Δ*Hira*Δ*Cdk8* ESCs cultured in 2i medium. B4 and E3 correspond to biological replicates. **b** Non-linear curve fitting of the proliferation curves shown in a using the exponential growth equation, plotted on a semi-logarithmic scale, the slope of the lines indicate rate of growth. The effect of the cell type (XX vs XO) was evaluated using a linear mixed-effects model, the analysis revealed that cell type did not have a statistically significant effect on the growth rate with a p value of 0.123 for the effect of the cell type XO. **c** Schematic depiction of the possible XO generation pathways. The first possibility results in the generation of one XO cell from one XX cell division, this would correspond to a gr value of 1 in the iterative model. The second possibility results in the generation of 2 XO cells from one XX cell division and corresponds to a gr value of 2 in the iterative model. **d** Iterative equations used to model the X chromosomal loss rate, pd corresponds to the proliferative difference of the XO over the XX cells, gr models how the XO cells arise as described in c, lr is the loss rate indicating the percentage of XX cells becoming XO per cell division. **e** Graphs produced by numerically evaluating the iterative model over a range of values to determine the ones best matching the experimental observations, the plotted curves show the change of the XO ratio over time, the red overlaid dots correspond to the experimentally determined XO ratios and time points. Numbers above the plots indicate the parameter values corresponding to the individual plots. **f** Description of the system of ODEs used to determine the X chromosomal loss rate. **g** Parameter estimation by curve fitting of the ODE model to experimentally determined points (indicated in red). Best fit corresponds to a loss rate of 0.3% with an R-squared value of 0.998021

The rapid loss of X chromosomes contrasts the high stability of autosomes in Δ*Hira*Δ*Cdk8* ESCs in 2i conditions. It also raised the question whether a proliferative advantage of XO cells exists that could explain an apparent X chromosomal instability by selection in culture. For this we considered two potential mechanisms. Firstly, we consider that the probability for losing autosomes and X chromosomes is equal, and subsequently XO ESCs would have a proliferative advantage through which their accumulation in 2i culture would be explained. Secondly, a specific mechanism would become active in naive Δ*Hira*Δ*Cdk8* ESCs that would lead to an increased probability of losing one X chromosome compared to autosomal loss frequency. The models differ by their assumptions on chromosome loss rates, whereby the latter model would require different loss rates for the X chromosome and autosomes. To experimentally investigate if one model could be ruled out we measured the proliferation rates for XX and XO Δ*Hira*Δ*Cdk8* ESCs in 2i medium, which are informative of the selection component. For this XX and XO ESCs were grown. Subsequently, we plated equal cell numbers into three 12-well plates with 2i medium and determined the cell numbers per well during the following 3 days. This allowed to determine the proliferation of ESCs after plating using the first day as a reference point to avoid effects from cell passaging and plating efficiency. Our data show that between day 1 and 2 after plating the cell number of XX and XO cells increased by a factor of 3.5 and 3.3, respectively (Fig. 5a, b). Conversely, between day 2 and 3 the increase of XX and XO ESCs was 2.4 and 2.9, respectively. From this we obtain doubling times of 13.7 and 14.2 h for XX and XO ESCs, respectively, from day one to day two. Longer doubling times of 20.2 and 15.8 h from day 2 to day three, and average doubling times of 16 and 14.8 h from day one to day three. Therefore, XO Δ*Hira*Δ*Cdk8* ESCs have at most a modest 1.2 fold proliferation advantage over XX Δ*Hira*Δ*Cdk8* ESCs in 2i culture (Fig. 5b). Statistical testing using mixed-effects models and ANOVA confirm a slight proliferative advantage of the XO cells which does not reach statistical significance (*p* = 0.2831).

We next thought to estimate the frequency of X chromosome loss per cell division from our X chromosome loss kinetics through numeric approaches. As the mechanism of X chromosome loss remains unknown we considered two possibilities (Fig. 5c). Firstly, non-disjunction events would generate one XO cell and one cell with 3 X chromosomes. Under the assumption that ESCs with 3 X chromosomes do not accumulate in culture, such an event would be considered to produce a single XO cell. Secondly, a loss event might eliminate one X chromosome before or during division and, hence, yield two XO cells. For the mathematical modelling, we perform iterative calculation of the numbers of XX and XO cells, as well as the XO ratio for each cell division (Fig. 5d) using a Python script. The proliferation of XX and XO cells, and the time points with known XO ratios are experimentally measured parameters. For determining the chromosome loss frequency we evaluate our model numerically over a range of values to determine the ones best matching the experimental observations. Using an experimentally determined proliferative advantage of at most 1.2 as an upper bound for the XO cells compared to the XX cells, we estimate an X chromosome loss rate between 0.6% and 0.8% for a mechanism that one XO cell arises from one XX cell. Conversely, for a mechanisms that two XO cells arise from one XX cell a slightly lower loss rate between 0.3% to 0.4% is obtained (Fig. 5e).

We next validate our calculation using a system of ordinary differential equations (ODEs) for calculating the XO ratio over time (Fig. 5f). We use our experimentally determined XO ratios at known time points for parameter estimation by curve fitting, yielding loss rates between 0.3% and 0.8% with a best fit produced by a 0.3% loss rate (R-squared = 0.998021) (Fig. 5g).

Typical eukaryotic chromosome loss rates in karyotypically stable cells are expected to be in the order of 10^–6^ per chromosome per cell division (Jelenić et al. 2018; Luo et al. 2000). Furthermore, our observation that chromosome 8 and 11 are stably maintained even during prolonged cultured in 2i of up to 18 days, suggests that there is no overall chromosomal instability in naive female Δ*Hira*Δ*Cdk8* ESCs. Our observed X chromosome loss rate, thus exceeds reported autosomal loss rates by 3 orders of magnitude and approximates that of chromosome instability (CIN) in colorectal cancer cells of 10^–2^ per chromosome per cell division, which is the highest rate that we are aware of being reported in the literature (Lengauer et al. 1997). In contrast to CIN colorectal cancer, our data on stable maintenance of two copies of chromosome 8 and 11 rule out that Δ*Hira*Δ*Cdk8* ESCs in 2i culture have acquired chromosome instability. Of further note, the drastically different loss rates are not confounded by effects of cell selection as these are fully accounted for by experimental proliferation measurements in our calculation. We propose a mechanism for elimination of one of the two X chromosomes as an explanation for the high X chromosome loss rate in Δ*Hira*Δ*Cdk8* ESCs in 2i culture.

### Induction of *Xist* rescues X chromosome instability in naive Δ*Hira*Δ*Cdk8* ESCs

Female mouse ESCs have not yet initiated XCI and possess two active X chromosomes. At this developmental stage, reactivation of the paternally inherited X chromosome would be observed in the epiblast cells of the blastocyst. It is unknown if a counting and choice mechanism becomes already operative before XCI. Moreover, mutation of *Hira* and *Cdk8* might potentially change aspects of the early stages of mammalian dosage compensation. For obtaining initial insight into the relation between dosage compensation and X chromosome elimination and further constrain a potential mechanism, we took advantage of the inducible *Xist* expression system in our Δ*Hira*Δ*Cdk8* ESCs. An inducible promoter is inserted upstream of the *Xist* gene on the TX X chromosome that allows for induction of *Xist* expression in the presence of Doxycycline (Dox) through a nls-rtTA transactivator expressed from the ROSA26 locus on chromosome 11 (Monfort et al. 2015).

In order to investigate the effect of *Xist* expression on X chromosomal stability, Δ*Hira*Δ*Cdk8* ESCs were cultured in SL medium and then split and grown in SL, SL + Dox, 2i and 2i + Dox medium, and X chromosomal status was assessed after 8, 18 and 28 days. We confirmed *Xist* expression after addition of Doxycycline by RNA FISH analysis. *Xist* clusters were observed in ESCs after addition of Doxycycline, whereas in untreated controls only a pinpoint signal was detected (Fig. 4c). Quantification of *Xist* signals (Fig. 4b) showed an increase of *Xist* clusters in the SL + Dox (62%, 109/176) and 2i + Dox (37%, 65/178) conditions. In contrast, the SL (6%, 9/142) and 2i (4%, 4/94) conditions show few *Xist* clusters and predominantly pinpoint signals. We next counted the number of X chromosomes using chromosome painting DNA FISH analysis after 8, 18 and 28 days in the different media conditions. We observed high X chromosomal stability in both independent Δ*Hira*Δ*Cdk8* double mutants when cultured in SL medium (Fig. 4a). After 8 days of culture 97% (56/58) and 96% (74/77) Δ*Hira*Δ*Cdk8* ESCs of double mutant replicate 1 and 2, respectively, maintained two X chromosomes, after 18 days 97% (65/67) and 95% (62/65) and after 28 days 97% (70/72) and 94% (64/68). Δ*Hira*Δ*Cdk8* ESCs cultured in SL + Dox medium showed similar levels of X chromosomal stability with 93% (63/68) and 93% (57/61) of analysed cells maintaining two X chromosomes after 8 days, 97% (58/60) and 97% (62/64) after 18 days, 96% (50/52) and 95% (63/66) after 28 days. This result is consistent with our earlier observation of X chromosome stability in SL medium and demonstrates that Doxycycline alone had no effect on X chromosomal stability. As expected Δ*Hira*Δ*Cdk8* ESCs that were cultured in 2i medium showed drastic loss of one X chromosome over time, with the 2X fraction decreasing from 69% (63/91) and 75% (58/77) after 8 days to 6% (5/86) and 11% (12/110) after 18 days and finally reaching 0.93% (1/107) and 1.75% (2/114) after 28 days in culture.

In contrast, Δ*Hira*Δ*Cdk8* ESCs cultured in 2i medium with Dox showed a substantial rescue of X chromosomal stability. The 2X fraction in the 2i + Dox condition changed from 95% (61/64) and 96% (48/50) at day 8 to 92% (103/112) and 93% (99/106) at day 18, to 92% (94/102) and 90% (82/91) at day 28. Therefore, *Xist* induction had caused a dramatic and significant rescue of X chromosomal stability from 6 to 92% and 11% to 93% at day 18 for the respective replicates and from 0.93% to 92% and 1.75% to 90% at day 28. We note that induction of *Xist* might have induced repression of X-linked genes on the TX X chromosome and conversely cells that might have lost the Cast/Ei X chromosome would become functionally nullisomic for X-linked genes and likely die. Therefore, our assay might be less sensitive for detecting X chromosome loss in 2i and Dox medium. However, considering that sensitivity of detection could be reduced up to twofold, whereas the frequency of cells maintaining two X chromosomes increased by a factor of over 10 suggests that lower sensitivity alone is unlikely to explain the rescue in X chromosome stability. Therefore, we conclude that induction of *Xist* has caused changes in the cell or triggered chromosomal changes that prevent elimination of one X chromosome in Δ*Hira*Δ*Cdk8* ESCs.

## Discussion

Our study discovers and characterizes an unexpected X chromosomal instability in hybrid female TX/Cast ESCs with mutations in the *Hira* and *Cdk8* genes. The high X chromosomal stability in our TX/Cast hybrid mESCs rules out a general instability of the cell line. Our finding that in Δ*Hira*Δ*Cdk8* ESCs X chromosome loss can be induced simply by a shift into 2i medium provides a model system for the investigation of chromosomal instability. This discovery allowed us to determine the kinetics of X chromosome loss with unprecedented precision. We show that X chromosome loss occurs in approximately 0.3% of Δ*Hira*Δ*Cdk8* ESCs per cell division and leads to rapid accumulation of the XO karyotype in the cultures. This chromosomal loss rate is in stark contrast with the observed overall high karyotypic stability of our hybrid mouse ESC line as well as our finding of stable autosomal maintenance in the Δ*Hira*Δ*Cdk8* ESC double mutant even during prolonged culture.

A potential explanation for the accumulation of XO cells could be through selection. From experimental measurements we obtain doubling times of 16 h for XX and 14.8 h for XO Δ*Hira*Δ*Cdk8* ESCs in 2i medium. While our statistical tests indicate that there is no significant difference in the growth rates of XX and XO cells, we use the modest experimentally determined proliferative advantage to model and calculate a lower bound for the expected X chromosomal loss rate. Notably, considering a 1.2 fold proliferation advantage for XO over XX cells as an upper bound in our calculations, we obtain a loss rate for the X chromosome in 2i cultured female Δ*Hira*Δ*Cdk8* ESCs that is 3 orders of magnitude higher than reported autosomal loss rates and approaches those observed in colorectal cancer cells. We have performed DNA FISH with chromosome 8 and 11 paint probes to measure autosomal stability. Both, trisomy 8 and 11 are karyotypic abnormalities, which are subject to positive selection in ESCs (Gaztelumendi and Nogués 2014; Kim et al. 2013). If the X chromosomal loss would occur with a similar frequency to that of autosomes and subsequently XO cells would be positively selected, we would expect the XO population to increase at a similar rate as cells with trisomy 8 or 11. Our experiments demonstrate that this is not the case and the majority of Δ*Hira*Δ*Cdk8* ESCs maintain two chromosomes 8 and 11. Based on the absence of a statistically significant proliferative advantage of the XO cells, the high X chromosomal loss rate calculated as a lower bound and the stable maintenance of autosomes, we therefore propose a chromosome elimination mechanism to explain the high observed X chromosome loss rates in 2i cultured female Δ*Hira*Δ*Cdk8* ESCs.

### Culture and genetic requirements of X chromosome elimination

Our study shows that X chromosome loss depends on specific genetic prerequisites and is strongly influenced by the culture medium. Δ*Hira*Δ*Cdk8* double mutant ESCs maintain two X chromosomes stably when cultured in SL medium, but show almost complete X chromosomal loss in the 2i medium. The finding that *Cdk8* and *Hira* single mutant ESCs cultured in 2i, while not as stable as in SL medium, still show high X chromosomal retention, suggests a synergistic effect of both mutations in combination with the effects of the MAPK and GSK3 inhibition. The observation that TX/Cast wild type ESCs show a stable X chromosomal constitution independent of the media condition further excludes the possibility of an inherent karyotypic instability.

The high X chromosomal loss rate in 2i raises some interesting questions concerning the formulation of this medium. While 2i is widely used in ESC cell culture (Tamm et al. 2013), concerns have been raised regarding the hypomethylation induced by a combination of PD0325901 and CHIR99021 inhibitors (Habibi et al. 2013; Choi et al. 2017). As hypomethylation is a prominent effect of 2i medium, we wanted to investigate if hypomethylation alone is sufficient to induce X chromosomal instability in female Δ*Hira*Δ*Cdk8* ESCs. Addition of the DNMT1 inhibitor GSK3484862 (Azevedo Portilho et al. 2021) to SL culture medium did not trigger X chromosome loss, even after prolonged culture of 21 days. Therefore, it is unlikely that demethylating effects of the 2i medium alone are sufficient to trigger X chromosome elimination in Δ*Hira*Δ*Cdk8* ESCs. However, at this point we cannot exclude the possibility that demethylation might act in combination with other effects caused by the inhibition of GSK3 and MEK1. Our comparison of 2i and combined GSK3 and SRC inhibitor medium demonstrates that MEK1 inhibition is a crucial factor for X chromosome elimination. Conversely, our finding that MEK1 inhibition alone in PD medium causes substantial X chromosome loss indicates that MEK1 is sufficient for X chromosome elimination in Δ*Hira*Δ*Cdk8* ESCs. Our data therefore identify MEK1 inhibition as a distinct characteristic of X chromosome elimination in female Δ*Hira*Δ*Cdk8* ESCs.

### A mechanisms for X chromosome elimination

An important observation is that chromosomal instability only affects the X chromosome and not autosomes. Therefore, common causes for chromosomal instability and aneuploidy, such as mitotic defects arising from chromosome mis-segregation (Compton 2011; Santaguida and Amon 2015) or mitotic checkpoint failures (Kops et al. 2005) are unlikely be involved as these would affect the autosomes to the same degree as the X chromosomes (A. Lynch et al. 2024). Instead, our data suggest an X chromosome specific feature for inducing chromosomal instability. Although, it remains unclear which feature differentiates the X chromosome from autosomes in the context of chromosomal stability, the presence of the X inactivation centre (*Xic*), the microsatellite DXZ4 (Bansal et al. 2020), and the pseudoautosomal region (PAR) can be considered. These regions could, thus, be considered interesting candidates for potential X chromosomal elements of an elimination mechanism.

Our observation that induction of *Xist* rescues X chromosome stability in 2i cultured Δ*Hira*Δ*Cdk8* ESCs possibly provides further constraints for a potential mechanism. X chromosome loss might be related to dosage compensation problems due to two active X chromosomes as suggested by Rücklé et al. (Rücklé et al. 2023). Reduced MAPK signalling has also been reported in female ESCs with XX karyotype compared to XO cells (Schulz et al. 2014). However, we note that MEK1 inhibition is included in 2i medium, and elevated X-linked gene dosage also exists in 2i cultured wild type ESCs and SL cultured Δ*Hira*Δ*Cdk8* ESCs, all of which do not display X chromosome elimination. While there is a difference in the pluripotency state between cells cultured in 2i and SL, with the 2i cultured cells retaining a more homogeneous pluripotent state, that more closely resembles the inner cell mass from which they are derived (Tosolini and Jouneau 2016) compared to SL cultured cells, these differences would affect the cells in the same way independent of genotype. Although elevated X-linked gene dosage is insufficient to trigger X chromosome elimination, we cannot rule out that elevated X-linked gene expression is an additional contributing factor for X chromosome elimination in Δ*Hira*Δ*Cdk8* ESCs in combination with a specific ground state of pluripotency. At this time it remains unclear which elements of the X chromosome are targeted by an elimination mechanism and which defects lead to its elimination downstream of loss of *Hira* and *Cdk8*. Our cell system might be useful to further define the mechanism of chromosome elimination in future studies.

### Chromosome or genome elimination in animals

Genome and chromosome elimination has been observed in a number of organisms throughout a wide range of species. Mechanisms have evolved to either protect genomes from unwanted sequences or to eliminate partial or entire chromosomes. The latter is associated with the elimination of sex chromosomes from somatic tissue (Dedukh and Krasikova 2022; Smith et al. 2018). Notably, sex chromosome elimination occurs in members of the bandicoot family *Paramelidae* where one of the X chromosomes in females and the Y chromosome in males is eliminated in some somatic tissue as a form of extended and irreversible dosage compensation (Shevchenko et al. 2013; Hayman and Martin 1965).

Although, the relation to X chromosome elimination in marsupials remains unclear, our work established a system for studying X chromosome elimination in a tractable cell culture system. This system could also be useful for revealing the mechanism leading to an XO karyotype in inbred mouse derived ESCs. Our work identifies *Hira* and *Cdk8* as genetic and MEK1 as a molecular factor that contribute to X chromosomal instability and provides a starting point for future molecular exploration of contributing pathways. Conversely, our cell system might also be useful for devising improved culture conditions for ESCs for the investigation of processes such as X inactivation, which depend on the presence of two active X chromosomes and could give insights into disease associated with X chromosomal loss.

## Material and methods

### Allelic PCR

Allelic PCR was used to identify the presence of TX and Cast/Ei X chromosomes. The TX X chromosome carries the doxycycline inducible Tet operator while the Cast/Ei X chromosome does not. PCR with primers 804 (AACCACGGAAGAACCGCAC) and 805 (CCAAGGTATGGAGTCACCAGG) over the region including the Tet operator, results in two DNA fragments of different sizes and allows the distinction between the two X chromosomes.

### Single colony PCR screen

The desired cells were grown according to standard culture practices in SL medium, then 800 cells were transferred into a 10 cm dish. Once colonies originating from single cells have grown to an appropriate size, they were picked and transferred to a 96 well plate. After becoming confluent again, the cells were split into two plates, one of which was frozen for future use, while the cells in the second plate are grown and then genotyped using the allelic PCR previously described. The genotyping data was then used to identify colonies retaining both X chromosomes. The identified XX colonies were thawed from the frozen plate and grown in the SL condition. After the cells have been grown to sufficient numbers, they were split and grown in the different media conditions. Once the desired passage number has been reached, 800 cells per media condition were transferred to a 10 cm dish, colonies were picked, cultured in 96 well plates, split, frozen and genotyped by allele specific PCR.

### Cell culture

The female hybrid TX/Cast cells, along with their mutant derivatives, were cultivated in accordance with previously outlined protocols (Postlmayr et al. 2020). Concisely, ESCs were grown on gelatine-coated dishes. ESC base medium consists of 500 mL DMEM(Gibco, 2,662,125), 75 mL fetal bovine serum (Biowest, S1810-500), 5 mL Non-Essential Amino Acids (Gibco, 11,140,050), 5 mL Sodium Pyruvate (Gibco, 11,360,070), 5 mL penicillin/streptomycin (Gibco, 2,076,694), 4 μL β-mercaptoethanol (M6250).

### Metaphase DNA FISH

TX/Cast hybrid ES cells were maintained in ESCs medium as described. To prepare metaphase chromosome spreads, cells were grown in medium containing 0.1 μg/mL of Colcimid solution (Merck, 477–30-5) for 3 h. Cells were washed with PBS and trypsinized with TrypLE (Gibco, 12,605,010). Cells were centrifuged at 200 g for 5 min and the supernatant discarded, 10 mL of hypotonic solution (DMEM, water 1:1) was added to the tube and incubated at 37 °C for 20 min. After incubation, the cells were again centrifuged at 200 g and the hypotonic solution was removed. The cell pellets were resuspended in 10 mL of fixative (Methanol, acetic acid 3:1) and mixed by gentle tapping, then incubated 20 min on ice. After the incubation, the cells were centrifuged at 200 g for 5 min, the fixative was carefully removed and 10 mL of fresh fixative were added, the cells were again incubated for 20 min on ice. After 20 min, the cells were centrifuged at 200 g for 5 min, the supernatant was removed and fresh fixative was added, the amount of fresh fixative added in this step depends on the desired final concentration of cells. The cell solution was applied to a SuperFrost microscopy slide and air-dried. 8 µl of DNA FISH probe (MetaSystems XMP X Green, D-1420–050-FI; XMP X Orange, D-1420–050-OR; XMP 11 Orange, D-1411–050-OR; XMP 8 Orange, D-1408–050-OR) diluted 1:3 in Hybrisol VII was added to the slides, the slides were then covered with a coverslip and sealed with rubber cement. Samples were denatured by heating at 75 °C for 2 min on a heat block. The samples were incubated at 37 °C in a humidified chamber for at least 16 h. After incubation, the slides were first washed in 0.4X SSC (pH 7) at 72 °C for 2 min, then in 2X SSC, 0.05% Tween-20 (pH 7) at room temperature for 30 s, before being briefly rinsed in distilled water and air-dried. The slides were then counterstained with 10 μL of DAPI/Vectashield (Vector Laboratories, H-1200). The coverslip was added and sealed with nail polish. Slides were analysed on a Zeiss Z1 microscope equipped with an X-cite 120 illuminator, images were taken with a connected Hamamatsu C11440 ORCA-Flash digital camera.

### RNA FISH

Cells were washed 2 times in PBS, trypsinized, harvested and diluted to approximately 200′000 cells/mL. The Cytospin funnel, bracket and slide were assembled and 250 µL of cell suspension were added to the desired wells. The assembly was centrifuged at 800 rpm for 3 min. The slides were removed from the bracket and rinsed in PBS at room temperature. The slides were then washed in CSK buffer at room temperature for 30 s, in CSK (100 mM NaCl, 300 mM sucrose, 3 mM MgCl2, 10 mM PIPES pH 6.8) + 0.5% Triton X100 at room temperature for 2 min and again in CSK buffer at room temperature for 30 s. The slides were then placed in 4% PFA at room temperature for 10 min before being rinsed in 70% Ethanol. The cells were then dehydrated by placing the slides in 70%, 80%, 95% and 100% ethanol for 2 min each. After dehydration the slides were air dried and the cell quality and density checked under a light microscope. 4 µL of *Xist* probe were added to the slide, covered with a coverslip and sealed with rubber cement, the slides were then incubated overnight in a humidified, dark chamber at 37 °C. On the next day, the coverslip was carefully removed by floating it in 4X SSC. The slides were then washed in 50% 2X SSC and 50% formamide at 39 °C with slight shaking in the water bath. The formamide was removed and the slides were washed 3 times in 2X SSC for 5 min each at 39 °C. The slides were washed in 1X SSC for 10 min at room temperature and air dried. The slides were counterstained with 10 µL DAPI/Vectashield, a fresh coverslip was added and sealed with nail polish. Slides were analysed on a Zeiss Z1 microscope equipped with an X-cite 120 Illuminator, images were taken with a connected Hamamatsu C11440 ORCA-Flash digital camera.

### Western blot

Whole-cell lysates were extracted in NP-40 lysis buffer (150 mM NaCl, 50 mM Tris–HCl (pH = 8), 1 mM EDTA, 1% NP-40) supplemented with cOmplete protease inhibitor cocktail and incubated on a rotating wheel for 20 min at 4 °C followed by sonication for 10 s at 10% amplitude. After centrifugation at 17′000 g for 20 min and 4 °C, the protein-containing supernatant was collected and stored. Protein concentrations were measured using a Bio-Rad Protein Assay (Bio-Rad Assay Dye, 5,000,006). Protein concentrations were adjusted to 2 µg / µL. Samples were denatured at 95 °C for 5 min. Equal volumes were subjected to SDS-PAGE, and blotted on nitrocellulose membranes. The membranes were first washed for 5 min with TBS at room temperature on a shaker, then 2 times for 5 min each with TBST (10X TBS: 24 g/L Tris, 80 g/L NaCl, pH = 7.5; 1X TBS + 0.05% Tween 20) at room temperature on a shaker and blocked with TBST + 5% non-fat dried milk(2.5 g milk in 50 mL TBST) for 30 min at room temperature on a shaker. Cdk8 (Santa Cruz Biotechnology, sc-131155) diluted 1:500 in TBST + 5% non-fat dried milk and Hira (Merck, 04–1488) diluted 1:1000 in TBST + 5% non-fat dried milk primary antibodies were added to the membrane and incubated overnight at 4 °C on a shaker. The membranes were washed 3-times with TBST and incubated with HRP-coupled secondary antibody for 1 h at room temperature. The membrane was washed 2 times in TBST for 5 min each at room temperature on a shaker and washed once in TBS for 5 min at room temperature on a shaker. Then Membranes were imaged using the enhanced chemiluminescence method on a Bio Rad ChemiDoc system.

### Generation of mutant ESC lines

Cdk8 loss of function mutants were generated as previously describe in (Postlmayr et al. 2020). The Hira knockout strategy is described in Fig. 1a. Briefly, two guide RNAs targeting the region around the start codon on *Cdk8* and WD repeats on *Hira* (*Cdk8*: gRNA1, 5′-CCGGTCCCCACCGCGGCCCT-3′ and gRNA2, 5′-AAGTTGGTCGAGGCACTTAC3′; *Hira*: gRNA1, 5′-CGCACACAGTTCACACATGC-3′ and gRNA2: 5′-GTTGACCATGAACACCTCAT-3′) were inserted into PX330 vector (Addgene, 422,300) and PX458 (Addgene, 48,138) respectively. The cloned plasmids were confirmed by sanger sequencing using a primer targeting the U6 promoter. The corresponding vectors: *Cdk8* gRNA1/gRNA2 and *Hira* gRNA1/gRNA2 were co-transfected into TX/Cast ESCs together with mCherry (Addgene, 632,524), cells were sorted 48 h after transfection. Mutant ESCs were confirmed by PCR and Western blot.

### Data analysis and software tools

In this study, statistical tests were performed in RStudio 2024.04.2 Build 764 using R version 4.4.1. Statistical significance is reported as: ns (*p* > 0.05), * (*p* < 0.05), ** (*p* < 0.01), *** (*p* < 0.001). Iterative modelling was done in Python 3, the Matplotlib package was used for visualization. Differential equations were solved, plotted and curve fitted in Mathematica 13.3.

## Supplementary Information

Below is the link to the electronic supplementary material.

Supplementary file1 (PDF 2.26 MB)

## Data Availability

No datasets were generated or analysed during the current study.
