# Supplementary material for: Cdk8 and Hira mutations trigger X chromosome elimination in naive female hybrid mouse embryonic stem cells
Source: Chromosome Res. 2024 Oct 10;32(4):12. doi: 10.1007/s10577-024-09756-w (PMC11467062; doi:10.1007/s10577-024-09756-w)
Supplement: Supplementary file 1 — Supplementary file1 (PDF 2.26 MB) [file 10577_2024_9756_MOESM1_ESM.pdf]

a

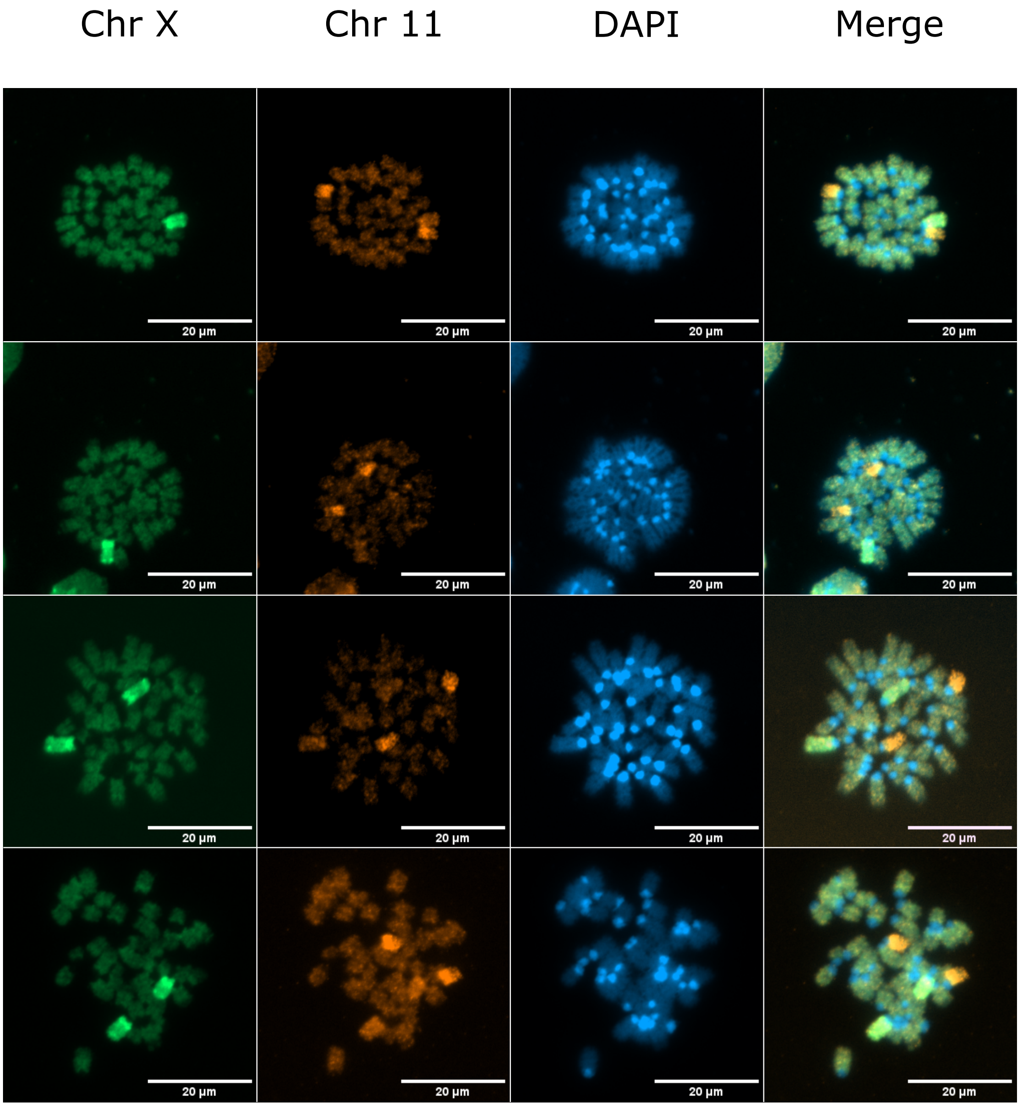

b

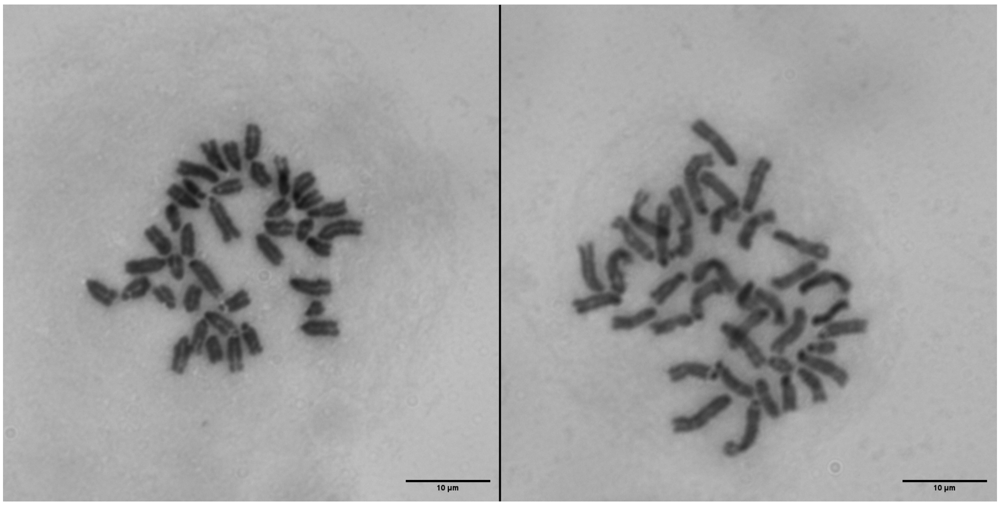

a Representative DNA FISH images with probes for chromosomes X (Green) and 11 (Orange), counterstained with DAPI (Blue). Scale bar corresponds to 20 µm. **b** Representative images of the 39 chromosome karyotype found in Δ*Hira*Δ*Cdk8* ESCs cultured in 2i medium. Scale bar corresponds to 10 µm.
